# Targeting keystone species helps restore the dysbiosis of butyrate‐producing bacteria in nonalcoholic fatty liver disease

**DOI:** 10.1002/imt2.61

**Published:** 2022-11-16

**Authors:** Dingfeng Wu, Lei Liu, Na Jiao, Yida Zhang, Li Yang, Chuan Tian, Ping Lan, Lixin Zhu, Rohit Loomba, Ruixin Zhu

**Affiliations:** ^1^ National Clinical Research Center for Child Health, The Children's Hospital Zhejiang University School of Medicine Hangzhou Zhejiang People's Republic of China; ^2^ The Shanghai Tenth People's Hospital, School of Life Sciences and Technology Tongji University Shanghai People's Republic of China; ^3^ Guangdong Provincial Key Laboratory of Colorectal and Pelvic Floor Diseases, The Sixth Affiliated Hospital, Guangdong Institute of Gastroenterology Sun Yat‐sen University Guangzhou People's Republic of China; ^4^ Department of Biomedical Informatics Harvard Medical School Boston Massachusetts USA; ^5^ State Key Laboratory of Biotherapy, West China Hospital Sichuan University and Collaborative Innovation Center Chengdu Sichuan People's Republic of China; ^6^ Department of Colorectal Surgery The Sixth Affiliated Hospital of Sun Yat‐sen University Guangzhou People's Republic of China; ^7^ Department of Pediatrics, Digestive Diseases and Nutrition Center The State University of New York at Buffalo Buffalo New York USA; ^8^ Department of Medicine, Division of Gastroenterology and Epidemiology, NAFLD Research Center University of California San Diego La Jolla California USA; ^9^ Research Institute GloriousMed Clinical Laboratory Co., Ltd. Shanghai People's Republic of China

**Keywords:** causal inference, dynamic intervention simulation, gut microbiota, keystone species, nonalcoholic fatty liver disease

## Abstract

The dysbiosis of the gut microbiome is one of the pathogenic factors of nonalcoholic fatty liver disease (NAFLD) and also affects the treatment and intervention of NAFLD. Among gut microbiomes, keystone species that regulate the integrity and stability of an ecological community have become the potential intervention targets for NAFLD. Here, we collected stool samples from 22 patients with nonalcoholic steatohepatitis (NASH), 25 obese patients, and 16 healthy individuals from New York for 16S rRNA gene sequencing. An algorithm was implemented to identify keystone species based on causal inference theories and dynamic intervention simulation. External validation was performed in an independent cohort from California. Eight keystone species in the gut of NAFLD, represented by *Porphyromonas loveana, Alistipes indistinctus*, and *Dialister pneumosintes*, were identified, which could efficiently restore the microbial composition of the NAFLD toward a normal gut microbiome with 92.3% recovery. These keystone species regulate intestinal amino acid metabolism and acid–base environment to promote the growth of the butyrate‐producing Lachnospiraceae and Ruminococcaceae species that are significantly reduced in NAFLD patients. Our findings demonstrate the importance of keystone species in restoring the microbial composition toward a normal gut microbiome, suggesting a novel potential microbial treatment for NAFLD.

## Highlights

- The dysbiosis of butyrate‐producing bacteria is a critical factor contributing to the development of nonalcoholic fatty liver disease (NAFLD).

- Causal algorithm intergraded with ecological theory and dynamic intervention simulation could mine microbial keystone species from metagenomic data.

- Keystone species of nonalcoholic steatohepatitis, such as *Porphyromonas loveana, Alistipes indistinctus*, and *Dialister pneumosintes*, provided potential precise intervention strategies for NAFLD treatment.

## Graphical abstract

In this study, we applied an algorithm to the keystone species identification in the gut microbiome, based on current causal inference theories and the dynamic intervention simulation. We identified the nonalcoholic steatohepatitis (NASH) keystone species combination, represented by *Porphyromonas loveana, Alistipes indistinctus*, and *Dialister pneumosintes*, that showed the highest potential for the microbial intervention of NASH.

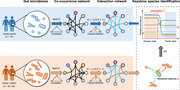

## INTRODUCTION

Nonalcoholic fatty liver disease (NAFLD) is a complex multifactorial disease whose pathogenesis remains unclear. The recent definition for metabolic‐associated fatty liver disease highlighted NAFLD as a continuum spanning from obesity to metabolic syndrome and diabetes, and the advanced form of NAFLD with hepatic inflammation is nonalcoholic steatohepatitis (NASH) [1]. Studies have shown that dysbiosis in the gut microbiota affects the initiation and development of NAFLD [2–4]. With the whole genome sequencing data of the gut microbiome, we have identified 37 microbial markers that can distinguish between mild and severe NAFLD patients [5]. In addition, we and others observed that microbe‐derived metabolites, including endogenous ethanol [6], bile acids [7–9], and amino acids [10, 11], contribute to the pathogenesis of NAFLD. Many microbial intervention therapies targeting the gut microbiota, such as prebiotics, probiotics, and fecal microbiota transplantation (FMT), are being considered to treat NAFLD [12]. However, these microbial interventions have not achieved a satisfactory effect [13–15], which may be partly explained by the structural complexity of the ecosystem we have in our gut. Therefore, further studies of the dynamic changes in the microbiome during disease development and the mechanisms behind the changes are essential for the identification of microbial targets in the precise treatment of NAFLD and other diseases related to the gut microbiome.

Keystone species represent excellent candidate targets for gut microbiome‐based interventions [16–19], as they are defined as the species required for the integrity and stability of the ecological system. Studies on keystone species have been conducted in several diseases, such as periodontal disease [20] and *Clostridium difficile* infection [21]. The alteration of the keystone species could affect much of the entire community through the interactions among the members of the ecosystem [22–24]. Thus, we hypothesize that, unlike FMT, the therapies targeted at keystone species could accurately control the state of the gut microbiome, which will be a potent precision strategy for NAFLD.

**Figure 1:**
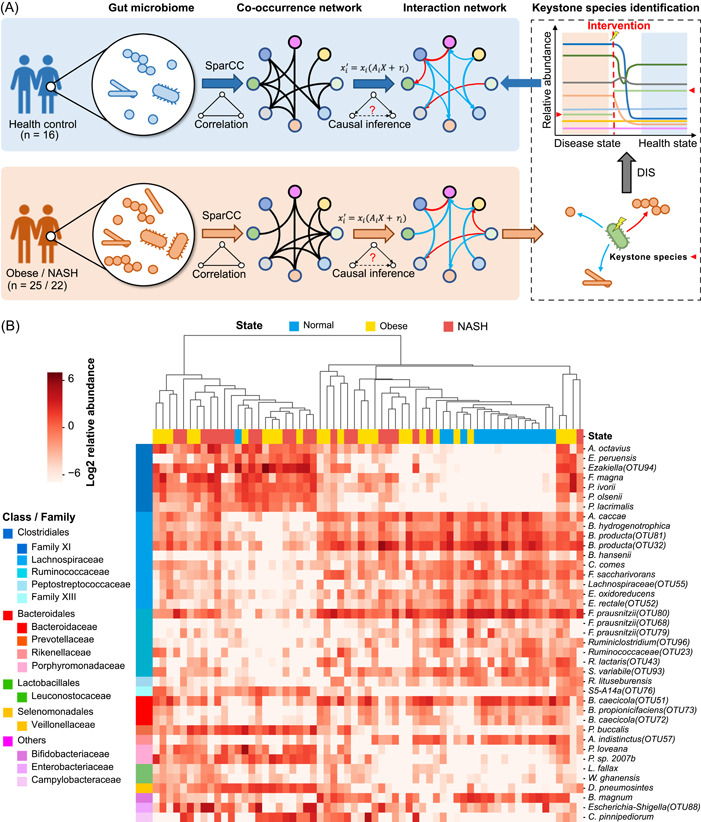
Illustration of the study concept and the abundant changes of the differential microbes. (A) Based on the abundance data and microbial co‐occurrence network, the causal inference algorithm intergraded with the ecological theory was utilized to construct the dynamic model of the microecosystem. Then, keystone species that could help restore the dysbiosis of the gut microecosystem were identified through dynamic intervention simulation (DIS). (B) Thirty‐nine species exhibited different abundance between normal controls and nonalcoholic steatohepatitis (NASH) patients, and a majority of these differential species belonged to class Clostridiales. In NASH, the differential species of Lachnospiraceae and Ruminococcaceae were downregulated. Similar changes were also observed in obesity. A full list of species is available in Supporting Information: Table S2.

The next‐generation sequencing techniques allowed the study of the microbiome in actual environments. Correlation analysis has become a common approach to study the interaction in the microecosystem [25, 26], and based on the topology of the co‐occurrence network, hub nodes in the network were defined and regarded as the keystone species [18, 27]. However, affected by confounders, the co‐occurrence network could not accurately imply the real causation between microorganisms [17, 28]. These limitations can be addressed by causal inference analysis. We have noticed that the causal theory has been valued and developed in various research fields and has also been applied in microbial research [29–31]. Compared with correlation analysis, the causal theory is conducive to construct the dynamic model of the microecosystem and identify the keystone species [32, 33]. Here, we collected stool samples from 22 patients with NASH and 16 healthy individuals from New York for 16S rRNA gene sequencing (Supporting Information: Table S1). Causal algorithms intergraded with the ecological theory were used to construct the dynamic model of the microecosystem, and keystone species were then identified through dynamic intervention simulation (DIS) (Figure 1A). Based on this strategy, we were able to characterize the changes in the composition and interaction of the gut microecosystem, which could redelineate the potential role of the gut microbiome in NAFLD and define the potential targets for precision intervention. This strategy was also applied to the 25 patients with obesity and an external validation cohort from California for further comparison (Supporting Information: Table S1).

## RESULTS

### Abundance changes of the gut microbial species in obesity and NASH

This study recruited 16 healthy controls, 25 obese patients, and 22 NASH patients. The quantitative abundance of the gut microbiome was obtained by 16S rRNA gene sequencing. Based on the causality theory, the dynamic models of the microecosystem of control and disease were constructed. Then, the keystone species of NASH were identified by DIS to guide the treatment of NASH (Figure 1A).

Compared with normal controls, the differential analysis identified 39 differential species in the gut of NASH patients (Figure 1B and Supporting Information: Table S2). These differential species mainly belonged to class Clostridiales, including Family XI, Lachnospiraceae, Ruminococcaceae, Peptostreptococcaceae, and Family III families. In NASH, the differential species of Family XI were upregulated, while those of Lachnospiraceae and Ruminococcaceae were downregulated (Figure 1B and Supporting Information: Figure S1A and Table S2). Similar changes were also observed in obesity. Interestingly, the gut microbiota exhibited a gradual change in the abundance of the differential species during the disease development from normal to obese and then to NASH (Supporting Information: Figure S1B). In addition, these differential species possessed strong discrimination abilities for normal and NASH samples (Supporting Information: Figure S1B); for example, *Porphyromonas loveana, Alistipes indistinctus (operational taxonomic unit [OTU]57)*, and *Dialister pneumosintes* achieved an area under the receiver operating characteristic curve (AUC) of 0.794, 0.766, and 0.832, respectively.

### Distinct patterns of the microbial interactions in the gut of normal, obese, and NASH subjects

**Figure 2:**
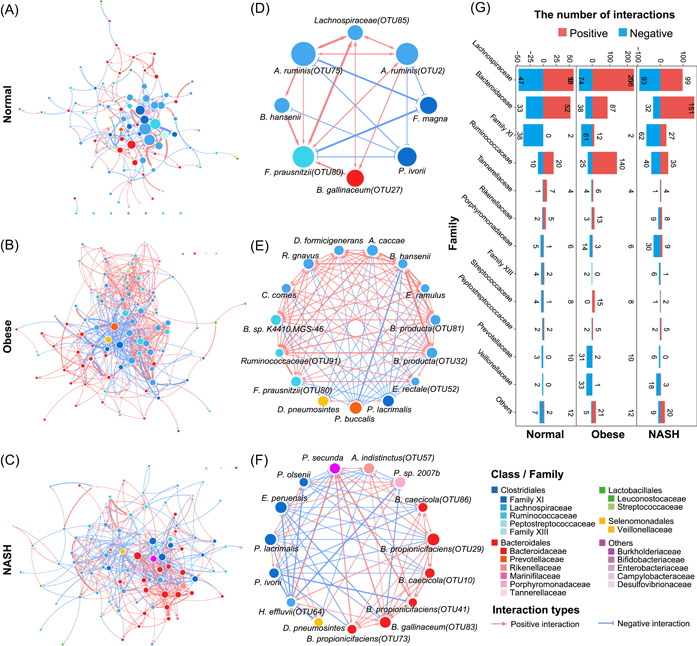
Microbial interaction networks and hub species in the gut of normal, obese, and nonalcoholic steatohepatitis (NASH) subjects. Microbial interaction networks of normal (A), obese (B), and NASH (C) study groups and the corresponding subnetworks of hub species of normal (D), obese (E), and NASH (F) groups were shown. The colors of nodes indicated the taxonomy of the species and the colors of edges indicated positive (red) or negative (blue) interaction between every two species. (G) Distribution of interactions for every family in microbial interaction networks of normal, obese, and NASH. A full list of species is available in Supporting Information: Table S2.

Microbial interaction networks in the gut of the normal, obese, and NASH subjects were constructed using a causal inference algorithm described in Section 5 (Figure 2A–C and Supporting Information: Figure S2), and the hub species of each study group were then identified based on the network topological properties (Figure 2D–F).

The gut microbiome of normal, obese, and NASH groups exhibited distinct patterns of microbial interactions (Figure 2). In normal subjects, the microbial interaction network exhibited a heterogeneous pattern (*R* ^2^ = 0.70 for power‐law distribution) [34], characteristics of a typical scale‐free network (Figure 2A). The hub species that belong to Lachnospiraceae (Positive [P]/Negative [N] = 58/47), Ruminococcaceae (P/N = 20/10), and Bacteroidaceae (P/N = 52/33) mainly imposed a positive impact, while those belonging to Family XI (P/N = 0/38) exerted a mainly negative impact on other microbes in the normal gut microbiome (Figure 2G and Supporting Information: Table S4).

Compared to normals, the microbial interactions in the gut of obesity and NASH exhibited immense alterations with relatively homogeneous patterns and more connections among the members of the microbial community (Figure 2B,C). Although the species of Lachnospiraceae, Family XI, and Bacteroidaceae still remained the hub species in the networks, more hub species including species of the families Prevotellaceae, Veillonellaceae, Peptoniphilus, Porphyromonadaceae, and so forth, were identified as hub species in obesity and NASH. The obesity and NASH‐specific hub species included *Prevotella buccalis, A. indistinctus, Porphyromonas sp. 2007b*, and *D. pneumosintes* (Figure 2D–F).

### Keystone species of NASH drive the changes of the diseased gut microbiome toward a normal microbiome

Alteration in the abundance of a keystone species is expected to induce changes in other species and profoundly impact intestinal homeostasis. Therefore, in this study, DIS was performed to simulate the dynamic alterations of the gut microbiome upon microbial intervention, and Iterative Feature Elimination (IFE) was conducted to identify those species that collectively exert the highest impact on the entire microbiome.

**Figure 3:**
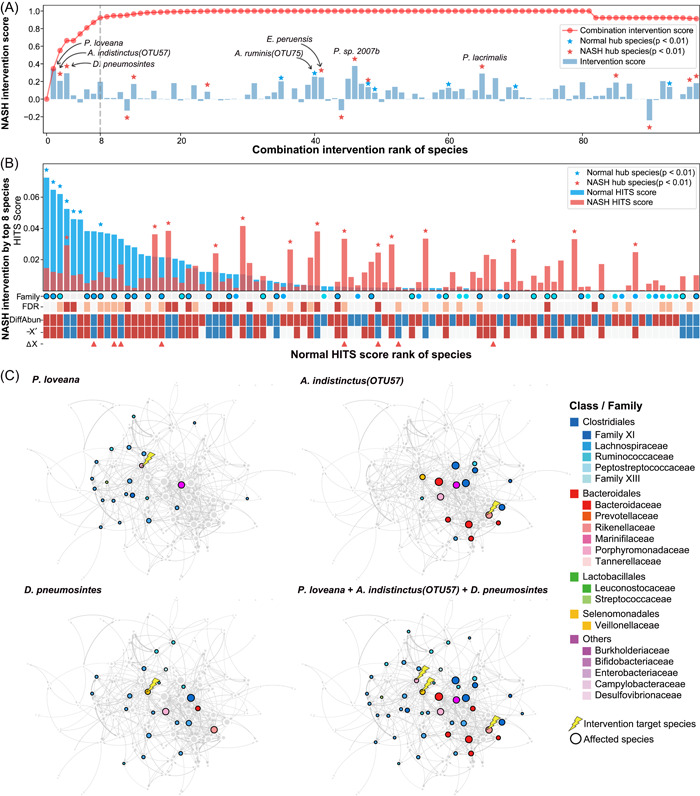
Dynamic intervention simulation (DIS) of the nonalcoholic steatohepatitis (NASH) microbiome. (A) Intervention scores (ISs) of the gut microbes. The IS of each microbe was shown in the bar plot and the stars with the blue and red color indicated the significance of the Hyperlink‐Induced Topic Search (HITS) score in the gut of the normals and the NASH, respectively. Negative IS indicated a change further away from the microbiome of healthy control. The red curve indicated the combination intervention scores (CISs) of the microbes sequentially selected by DIS, which represents the intervention effect after the simultaneous intervention of multiple species. The first eight keystone species, achieving a CIS greater than 0.9, were indicated by the dashed line. (B) Effect of microbial intervention on the NASH microbiome according to DIS with the Top 8 keystone species from (A). HITS scores of species in the normal microbiome were ranked in the bar plot, and the hub species were marked with stars. The species of Lachnospiraceae (blue) and Ruminococcaceae (light blue) were marked on the Family axis, with black borders indicating that the abundance recovered to normal levels after the intervention. DiffAbun: abundance change from normal to NASH (red: increase; blue: decrease), with the false discovery rate (FDR) indicated above (red: FDR < 0.01; light red: FDR < 0.05). $-X^{\prime }$: negative representation of instant microbial abundance changes upon the intervention (Red: $-X^{\prime }$ > 0; Blue: $-X^{\prime }$ < 0). Eight keystone species for intervention were marked by triangles in ∆X. (C) Intervention effects of the first three keystone species (*Porphyromonas loveana, Alistipes indistinctus*, and *Dialister pneumosintes*). Black borders of the nodes indicated significant intervention effects. A full list of species is available in Supporting Information: Table S2.

Consistent with their topologically central positions in the microbial interaction networks, the hub species were highly influential in the gut microbiome according to our DIS analyses (Figure 3A and Supporting Information: Figure S3A). Targeting hub species in obese patients, such as *P. buccalis* (intervention score [IS] = 0.397), *Ruminococcus torques* (IS = 0.288), *Blautia hansenii* (IS = 0.284), *Anaerostipes caccae* (IS = 0.281), and *D*. *pneumosintes* (IS = 0.279), was able to cause significant changes in the structures of the gut microbiome, toward restoring a normal gut microbiome (Supporting Information: Table S2). Similarly, with the NASH gut microbiome, targeting hub species such as *P*. *sp. 2007b* (IS = 0.376), *D*. *pneumosintes* (IS = 0.294), *Peptoniphilus lacrimalis* (IS = 0.289), *Agathobacter ruminis* (IS = 0.254), and *Ezakiella peruensis* (IS = 0.247) was able to cause significant changes in the gut toward restoring a normal gut microbiome (Supporting Information: Table S2). Targeting single species other than the hub species produced a negligible impact on the composition of the gut microbiomes (Supporting Information: Figure S3C,D).

Intervention targeting multiple species simultaneously would produce a better outcome than targeting a single species [17]. Therefore, we took a novel approach of integrating the DIS and IFE algorithms to identify the keystone species combinations that have the highest potential for microbial interventions. We identified 11 and 8 keystone species from the obese and NASH microbiomes, respectively. Combination intervention scores (CISs) of the identified keystone species combinations were 0.903 and 0.923, respectively Supporting Information: (Figure 3A and Supporting Information: Figure S3A and Table S2), suggesting that the microbiome in the gut of the patients can be maximally restored toward a normal microbiome by targeting these keystone species (Figure 3B and Supporting Information: Figures S3B). Targeting eight keystone species in the NASH caused changes in the species of Lachnospiraceae and Ruminococcaceae, which are the hub species in the normal microbiome, to increase toward the abundance found in the normal microbiome (Figure 3B). Meanwhile, hub species in the NASH microbiome also responded to the intervention, and most of the differential species in NASH responded to the keystone intervention with abundance changed toward the normal microbiome.

Among eight NASH keystone species, the first three keystone species, *P. loveana, A. indistinctus*, and *D. pneumosintes*, exhibited strong intervention capacity (CIS = 0.664), and the other five keystone species played minor roles (Figure 3A). Similarly, the first three keystone species of obesity exhibited strong intervention capacity (CIS = 0.587, Supporting Information: Figure S3A). The first three keystone species were hub species (*p* < 0.05) in the normal or diseased microbial network, and they caused major changes when they are targeted for microbial interventions in NASH patients (Figure 3C and Supporting Information: Table S5). *P. loveana* was elevated in NASH; therefore, we removed *P. loveana* as a microbial intervention. Removing *P. loveana* mainly increased the abundance of the Lachnospiraceae species, most of which were the hub species in the normal microbiome. *A. indistinctus* was decreased in NASH. Adding *A. indistinctus* mainly elevated species of Bacteroidaceae and reduced Family XI. *D. pneumosintes* was elevated in NASH. Removing *D. pneumosintes* increased the abundance of Lachnospiraceae and Ruminococcaceae species. Intervention with these three species simultaneously could restore the abundance of many gut microbes, especially the species of Lachnospiraceae and Ruminococcaceae (both being abundant families in normal microbiomes), thereby promoting the reconstruction of a normal intestinal microbiome (Figure 3C).

### Potential mechanisms for the keystone species to impact the NASH microbiome

**Figure 4:**
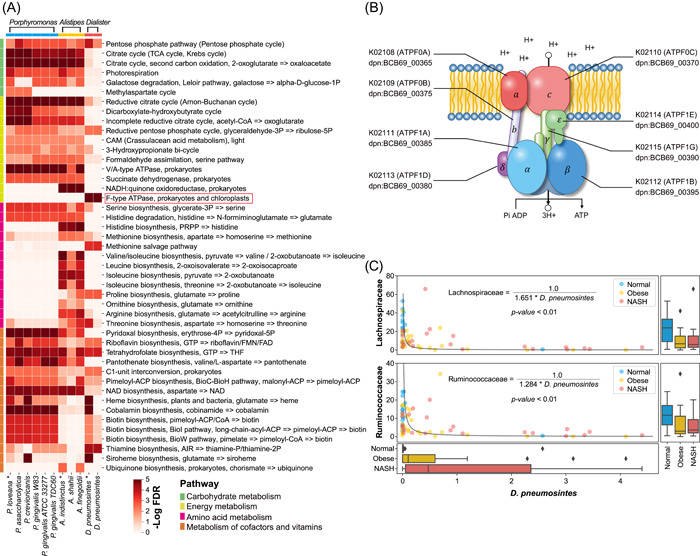
Functional enrichment analysis on keystone species. (A) Species *Porphyromonas loveana, Alistipes indistinctus*, and *Dialister pneumosintes* were subjected to KEGG module enrichment analysis based on their gene (KEGG orthology, KO) annotations using PICRUST2. Modules were classified according to the KEGG pathways and marked with different colors. The heatmap plotted the significance of enrichment (‐log false discovery rate [FDR]). (B) *D. pneumosintes* encodes the entire eight genes for the F‐type ATPase complex. This protein complex can utilize the membrane proton gradient for ATP production. (C) Abundance change of *D. pneumosintes* was significantly correlated to the abundance changes of Family Lachnospiraceae and Ruminococcaceae, fitting with a reciprocal function in both cases. The box plots at the axis are the abundance distribution of Lachnospiraceae, Ruminococcaceae, and *D. pneumosintes* in the gut of normal, obese, and nonalcoholic steatohepatitis subjects.

*P. loveana, A. indistinctus*, and *D. pneumosintes* were identified as the top keystone species in the NASH microbiome and exhibited the highest capabilities for the intervention of the NASH microbiome. To understand the mechanisms behind the massive alterations in the microbiome induced by targeting these keystone species, we obtained their genome information with PICRUST2 [35] and performed the functional (KEGG Module) enrichment analysis (Figure 4A and Supporting Information: Table S6).

As shown in Figure 4A, the genome of *P. loveana* is severely deficient in amino acid‐producing genes while rich in cofactor and vitamin‐producing genes. Amino acids, such as glutamate, glycine, alanine, tyrosine, aspartate, valine, and so forth, are required substrates for the production of cofactors and vitamins [36, 37] and may be used for the production of other molecules including short‐chain fatty acids (SCFAs) [38]. Therefore, the increased abundance of *P. loveana* would lead to enhanced amino acid consumptions in NASH (Supporting Information: Figure S4). A similar gene enrichment pattern was observed for the keystone *D. pneumosintes*, indicating that *D. pneumosintes* may contribute to elevated amino acid consumption in NASH.

Keystone species *A. indistinctus* participated in the production of a variety of amino acids including serine, threonine, valine, isoleucine, leucine, arginine, proline, glutamate, and histidine (Figure 4A). The downregulation of *A. indistinctus* (false discovery rate [FDR] = 0.020, Supporting Information: Table S2) suggested reduced microbial synthesis of amino acids.

Importantly, *D. pneumosintes* encodes the entire eight genes for the F‐type ATPase complex (Figure 4A,B), implicating that *D. pneumosintes* can utilize H^+^ in the intestinal lumen to synthesize ATP. In our intervention simulation, targeting *D. pneumosintes* caused changes in the abundance of Lachnospiraceae and Ruminococcaceae species (Figure 3C and Supporting Information: Table S5). Consistently, correlation analyses showed that the abundance of *D*. *pneumosintes* was negatively correlated with those of Lachnospiraceae and Ruminococcaceae (*p* < 0.01, Figure 4C). Previous studies have shown that the Lachnospiraceae and Ruminococcaceae species are very sensitive to environmental pH, which seriously affects their abilities of butyric acid production [39, 40]. Therefore, the increased abundance of *D. pneumosintes* in NASH (FDR = 0.004, Supporting Information: Table S2) might change the intestinal pH and consequently decrease the abundance and butyric acid production of Lachnospiraceae and Ruminococcaceae.

In general, although low in relative abundance, *P. loveana, A. indistinctus*, and *D. pneumosintes* have regulatory effects on Lachnospiraceae and Ruminococcaceae (Supporting Information: Figure S5), supporting their crucial role in the NASH gut microecosystem.

### Validation of the method for keystone species identification with an independent NAFLD cohort

We observed a similar pattern of microbial change in the validation NAFLD cohort (Supporting Information: Table S3). Briefly, Lachnospiraceae and Ruminococcaceae species exhibited significantly reduced abundance in the gut of NAFLD patients (*p* < 0.01). In addition, *A*. *indistinctus*, a keystone species in the NASH microbiome in the discovery cohort, was also significantly downregulated (*p* = 0.01 in normal vs. NASH‐cirrhosis and *p* = 0.017 in normal vs. NAFLD without fibrosis, Supporting Information: Figure S6A,B and Table S3). These differential species also exhibited strong sample discrimination abilities with the highest AUC = 0.894 (Erysipelotrichaceae [OTU96] in normal vs. NASH‐cirrhosis, Supporting Information: Figure S6B).

Again, the Lachnospiraceae and Ruminococcaceae species were the hub species of the normal gut microbiota, playing key roles in maintaining the homeostasis of the normal microbial communities (Supporting Information: Table S3).

The keystone species identified from the validation cohort were partly overlapping with those identified from the discovery cohort. The common keystone species from both cohorts included *Blautia producta, Bacteroides barnesiae*, and *A. caccae* (Supporting Information: Table S3). Consistently, targeting these keystone species for microbial interventions was able to restore the abundance of Lachnospiraceae and Ruminococcaceae (Supporting Information: Figure S7).

## DISCUSSION

In this study, we applied an algorithm to the keystone species identification in the gut microbiome, based on current causal inference theories and the DIS. We identified the NASH keystone species combination, represented by *P. loveana, A. indistinctus*, and *D. pneumosintes*, that showed the highest potential for the microbial intervention of NASH.

The most outstanding characteristic of the gut microbiome in both adolescent (discovery cohort) and adult (validation cohort) NAFLD seemed to be decreased abundance in Lachnospiraceae and Ruminococcaceae, two dominant families in Clostridiales. As the major butyrate‐producing bacteria in the intestine [41], Lachnospiraceae and Ruminococcaceae may play important roles in suppressing intestinal inflammation via the stimulatory effect of butyrate on T regulatory cells in the mucosa [42, 43] and consequently suppress the pathogenesis of NASH [44, 45]. The structural and functional importance of these two bacterial families makes them desirable targets in the microbial intervention of NAFLD.

With the NASH microbiome, the keystone species, especially *P. loveana, A. indistinctus*, and *D. pneumosintes*, could rapidly alter the abundance of Lachnospiraceae and Ruminococcaceae species and restore the microbial composition toward a normal gut microbiome (Supporting Information: Figure S8). These species are able to impact the other community members with fermentation products. *P. loveana* was elevated in NASH and therefore was removed from the NASH microbiome for intervention. Reduced abundance of *P. loveana* leads to decreased consumption of amino acids, therefore, leaving more resources for the growth of other bacteria including Lachnospiraceae and Ruminococcaceae. *A. indistinctus* was decreased in NASH and, therefore, was added to the NASH microbiome for intervention. *A. indistinctus* is equipped with many genes related to amino acid synthesis. In addition to supporting protein synthesis of the microbial communities, these amino acids produced in the gut may serve as microbial fermentation substrates for SCFA production [46], such as butyrate synthesis from threonine, lysine, and glutamate [47]. As such, the increased presence of *A. indistinctus* may promote not only the growth of other members of the microbial community with its amino acid production but also the intestinal balanced immunity with SCFA production [44, 45]. *D. pneumosintes* was elevated in NASH and therefore was removed from the NASH microbiome for intervention. *D. pneumosintes* encodes all eight genes of the F‐type ATPase complex that can use protons in the intestinal environment for ATP synthesis. Thus reducing *D. pneumosintes* may help maintain a low intestinal pH that promotes the growth of the butyrate‐producing Lachnospiraceae and Ruminococcaceae species [39, 40].

Compared to the keystone species with the top CISs, the abundance of Lachnospiraceae and Ruminococcaceae species were more profoundly altered in the NASH microbiome, yet they did not achieve the highest ISs in DIS and their performance in IFE was not impressive either. Our results indicate that the keystone species may not be the most abundant species in the microbial community and that targeting species mostly altered in diseases may not be an effective microbial intervention strategy. In contrast, *P. loveana, A. indistinctus*, and *D. pneumosintes*, the keystone species that out‐performed other keystone species in IFE, were not the top altered species in diseases, but they exhibited the highest potential in restoring a normal gut microbiome. Their ability for microbial interventions of NASH may be attributed to their metabolic products that have a profound influence on other members of the microbial community, and these broad influences allowed them special roles in maintaining the integrity and stability of the normal microbiome.

Although it is very difficult to reconstruct the interaction network of the complex system [33], especially for cross‐sectional data, we proposed a microecosystem modeling method based on causal inference, which could benefit from the prior knowledge and improve the accuracy of interaction discovery (Supporting Information: Figure S9) [48, 49]. Keystone species were then identified through DIS according to their definition. The rich prior knowledge and rigorous method framework ensured the reliability of this study and the feasibility of subsequent experiments and clinical treatment.

## CONCLUSION

In summary, we identified microbial keystones from cross‐sectional microbiome data by leveraging causal inference analysis and DIS. The identified keystone species in the gut of NAFLD, represented by *P. loveana, A. indistinctus*, and *D. pneumosintes*, could efficiently modulate the microbial composition of the NAFLD, especially Lachnospiraceae and Ruminococcaceae, toward a normal gut microbiome. Validated with an independent NAFLD cohort, our findings suggested a novel potential microbial treatment for NAFLD. Moreover, the described strategy for microbial keystone species identification may benefit microbiome studies in the broad fields of medicine, environmental science, and microbiology.

## METHODS

### Discovery cohort

Enrollment of the discovery cohort from New York was described in our previous study [6]. The raw sequencing reads and associated meta‐data were downloaded from MG‐Rast (https://metagenomics.anl.gov/linkin.cgi?project=1195). Briefly, the discovery cohort included fecal samples of adolescents collected from 22 biopsy‐proven patients with NASH, 25 obese patients, and 16 healthy controls (Supporting Information: Table S1) and were pyrosequenced on a 454‐FLX‐Titanium Genome Sequencer (Roche 454 Life Sciences). All NASH patients fulfilled Kleiner's criteria on hepatic fat infiltration, inflammation, and fibrosis through liver biopsy. Patients with body mass indices (BMIs) higher than the 95th percentile and normal liver function tests were recruited in the obese group. The healthy controls were volunteers whose BMI was less than the 85th percentile. In addition, the dietary intake (protein, fat, and carbohydrate) of all individuals was assessed by Dietary Instrument for Nutrition Education (DINE healthy, version 7.0.1) and the Centers for Disease Control (CDC) food frequency questionnaire. At the same time, all volunteers in this cohort were prohibited from drinking alcohol to ensure that alcohol intake would not become a confounding factor in this study.

### Validation cohort

External validation of the results was performed in an independent cohort we have previously published [50]. The raw sequencing data could be accessed through the European Molecular Biology Laboratory‐European Bioinformatics Institute (EMBL‐EBI, https://www.ebi.ac.uk/ena/data/view/PRJEB28350). This cohort included 31 healthy controls, 14 NAFLD patients without advanced fibrosis, and 24 NAFLD patients with cirrhosis (Supporting Information: Table S1) and was sequenced with Illumina MiSeq.

### Metagenomic analysis

Operational taxonomic units (OTUs) were de novo clustered at 97% sequence identity and chimeras were removed with Qiime2 after quality control of the 16S rRNA gene sequencing data [51]. Taxonomy classification was assigned using classify‐sklearn based on a Naive Bayes classifier against the Silva‐132‐99 reference sequences. OTUs that cannot be precisely annotated to any species were reassigned to species with the most similar sequences in the same genus (or family) by NCBI Blast with the default setting. And then, OTUs unassigned at the class level were removed for further analysis. Ninety‐seven and 190 OTUs were obtained with a sample coverage ≥0.5 from the discovery and validation cohorts, respectively.

### Differential abundance analysis and evaluation of the sample discrimination ability of microbes

The Wilcoxon rank‐sum test was performed to identify microbes with differential abundance between study groups, and the Benjamini–Hochberg was applied to control the FDR in multiple comparisons (FDR ≤ 0.05). These differential microbes were then individually evaluated for their ability in sample discrimination using the AUC.

### Keystone species identification

The pipeline mainly consists of two steps (Supporting Information: Methods, Movie S1): (1) a causal inference‐based method was implemented to construct the microbial causal interaction networks and (2) keystone species were identified with DIS based on microbial interactions (Supporting Information: Figure S8).

(1) Interaction network construction by causal inference
  Causal inference needs prior knowledge. Considering the compositional characteristics of microbial sequencing data, we used SparCC to calculate the co‐occurrences of the microbial species in the gut microbiome [52]. SparCC avoids the inaccuracies that usually occur with relative abundance in correlation analysis. Only relationships with *p* ≤ 0.01 (permutation test with 1000 permutations) were included in the prior network.
  Causal inference was performed by integrating two mainstream causal inference frameworks, Robins’ potential outcome (counterfactual) model [48] and Pearl's graphical model [49]. Our approach effectively controls the influence of confounders and infers causality more accurately on the microbial cross‐sectional cohort. We extended the application of causal inference to microbiome analysis with the generalized Lotka–Volterra (gLV) model, a well‐known dynamic model for the microbiome study [53]. Meanwhile, the iterative optimization strategy was used to improve the reliability of the prior network and the accuracy of the inferred causal relations. The permutation test was performed to determine significant causalities (*p* ≤ 0.01) (Supporting Information: Methods).
(2) Keystone species identification

By definition [19, 22–24], keystone species is one of the core driving factors to maintain or damage the integrity and stability of the microbial communities. Therefore, the keystone species of the microbiome represent effective intervention targets. Here, we applied DIS to evaluate the impact of targeting each candidate keystone species on the entire gut microbiome based on the microbial interactions so as to determine the keystone species that have the highest impact in the gut microbiome.

The topological importance of a species was a key indicator to evaluate its impact on other species in the entire ecosystem and thus the important basis in our keystone species identification. To assess the strength of the microbial interactions obtained by causal inference, Hyperlink‐Induced Topic Search (HITS), a network node importance evaluation algorithm [54] was used to quantify the influence of every species in the community [28]. The significance of HITS scores was assessed by the permutation test with 1000 permutations. Species with significantly higher HITS scores (*p* ≤ 0.01) were defined as hub species.

As reported previously [53], the gLV equation was applied to construct the dynamic model of microbial interactions and abundance changes in DIS. DIS was conducted to evaluate the abilities of targeting each candidate keystone species on restoring a normal microbial structure from a diseased microbiome. The performance of the intervention by each candidate species, the ISs, was adjusted by integrating the HITS scores of the species in the normal microbiome to reward the interventions that preferentially restored the species with greater topological significance in the normal microbial network. Finally, the IFE algorithm was used to determine the optimal combinations of the keystone species with the maximum CIS (Supporting Information: Methods).

### Microbial functional enrichment analysis

With the information on the gene annotations and the integrated modules from the KEGG database, functional enrichment analysis was performed to assess the biological functions of microbial species. The KEGG orthologies information of microbes was predicted via PICRUST2 [35]. The enrichment analysis of KEGG modules was conducted with one‐sided Fisher's exact test and adjusted with the Benjamini–Hochberg method. Modules with FDR ≤ 0.01 were considered significantly enriched modules.

### Statistical analysis

Statistical significance was determined by the two‐sided Wilcoxon rank‐sum test, permutation test, or one‐sided Fisher's exact test. When not specified otherwise, the statistical analyses have been performed with Python (3.6.0) and referenced in the description of the analyses. The number and description of the samples are reported in Supporting Information: Table S1, and the statistical analyses always refer to the whole set of samples in the specific condition of interest.

## AUTHOR CONTRIBUTIONS

Ruixin Zhu, Rohit Loomba, and Lixin Zhu conceived and designed the project. Each author has contributed significantly to the submitted work. Dingfeng Wu, Lei Liu, and Na Jiao drafted the manuscript. Yida Zhang, Li Yang, Chuan Tian, Ping Lan, Lixin Zhu, Rohit Loomba, and Ruixin Zhu revised the manuscript. All authors read and approved the final manuscript.

## CONFLICT OF INTEREST

The authors have declared no competing interest.

## Supporting information

Supporting information.

Supporting information.

Movie S1.

Supporting information.

Supporting information.

## Data Availability

No new sequencing data was used in this paper. The processed data have been uploaded to the NODE database (https://www.biosino.org/node/analysis/detail/OEZ013156). All the software packages used in this study are open source and publicly available, and the code used in this study is available on GitHub at https://github.com/tjcadd2020/NAFLD_keystone. Supplementary materials (figures, tables, scripts, graphical abstract, slides, videos, Chinese translated version, and update materials) may be found in the online DOI or iMeta Science https://www.imeta.science/.
