# Supplementary material for: Targeting keystone species helps restore the dysbiosis of butyrate‐producing bacteria in nonalcoholic fatty liver disease
Source: Imeta. 2022 Nov 16;1(4):e61. doi: 10.1002/imt2.61 (PMC10989787; doi:10.1002/imt2.61)
Supplement: Supplementary file 1 — Supporting information. [file IMT2-1-e61-s002.docx]

**Supplementary Figures**

**Figure S1. The abundance changes of the differential microbes and their sample discrimination abilities.** (A) The composition of the differential species in normal, obesity, and non-alcoholic steatohepatitis (NASH). (B) The abundance changes of the differential microbes and their sample discrimination abilities among normal, obesity, and NASH. A full list of species is available in Table S2.

**Figure S2. Microbial interactions between species.** Microbial interactions determined by different approaches (i.e., Spearman correlation, SparCC, and causal inference analysis) in normal, obesity, and non-alcoholic steatohepatitis (NASH). Each row and column in the heatmap represent a species, and the microbial interactions with *P* < 0.05 were displayed by colors (red: positive interaction; blue: negative interaction).

**Figure S3. Dynamic intervention simulation (DIS) of obesity microbiome.** (A) Intervention score (IS) of microbes in obesity. The IS of each microbe was shown in the bar plot and stars with the blue and yellow color indicated the significance of the Hyperlink-Induced Topic Search (HITS) score in normal and obesity, respectively. Negative IS indicated a change further away from the microbiome of a healthy control. The red curve indicated combination intervention scores (CIS) of the microbes sequentially selected by DIS, which represents the intervention effect after the simultaneous intervention of multiple species. The first 11 keystone species, achieving the combination intervention score > 0.9, were indicated by the dashed line. (B) Effect of microbial intervention on obesity microbiome according to DIS with the top 11 keystone species from (A). HITS scores of species were ranked in the bar plot, and hub species were marked with stars. The species of Lachnospiraceae (blue) and Ruminococcaceae (light blue) were marked in the Family axis. Nodes with black borders indicated that the abundance of species recovered to normal levels after intervention. DiffAbun: abundance change from normal to obesity (red: increase; blue: decrease), with false discovery rate (FDR) indicated above (red: FDR < 0.01; light red: FDR < 0.05). $-X^{'}$: negative representation of instant microbial abundance changes upon the intervention (red: > 0; blue: < 0). Eleven keystone species for intervention were marked by triangles in $\Delta X$. (C) Distribution of the intervention scores of the microbial species in obesity and (D) Non-alcoholic steatohepatitis. Here hub species were defined as species with *P* < 0.05 for HITS analysis. ** indicates *P* < 0.01 with the Wilcoxon rank-sum test. A full list of species is available in Table S2.

**Figure S4. Schematic diagram of the important metabolism processes of *Porphyromonas loveana*.**

**Figure S5.** **The correlation between the abundance changes of keystone species, and Family Lachnospiraceae, and Ruminococcaceae.** The abundance changes of (A) *Porphyromonas loveana* and (B) *Alistipes indistinctus* were significantly correlated to the abundance changes of Family Lachnospiraceae and Ruminococcaceae. The box plots at the axis are the abundance distribution of Lachnospiraceae, Ruminococcaceae, *P. loveana*, and *A. indistinctus* in the gut of normal, obese, and non-alcoholic steatohepatitis subjects.

**Figure S6. The differential abundance changes in the non-alcoholic fatty liver disease (NAFLD) cohort from California.** (A) The abundance heatmap of the differential species (*P* < 0.01) between normal and NAFLD-cirrhosis. (B) The abundance changes of differential microbes (*P* < 0.01) and their sample discrimination abilities between normal and NAFLD-cirrhosis. A full list of species is available in Table S3.

**Figure S7. The dynamic intervention simulation analysis in the non-alcoholic fatty liver disease (NAFLD) cohort from California.** (A) Intervention score of microbes in NAFLD-without advanced fibrosis (AF). (B) Effect of microbial intervention with keystone species in NAFLD-without AF. (C) Intervention score of microbes in NAFLD-cirrhosis. The first 28 keystone species, achieving the combination intervention score > 0.8, were indicated by the dashed line. (D) Effect of microbial intervention with keystone species in NAFLD-cirrhosis.


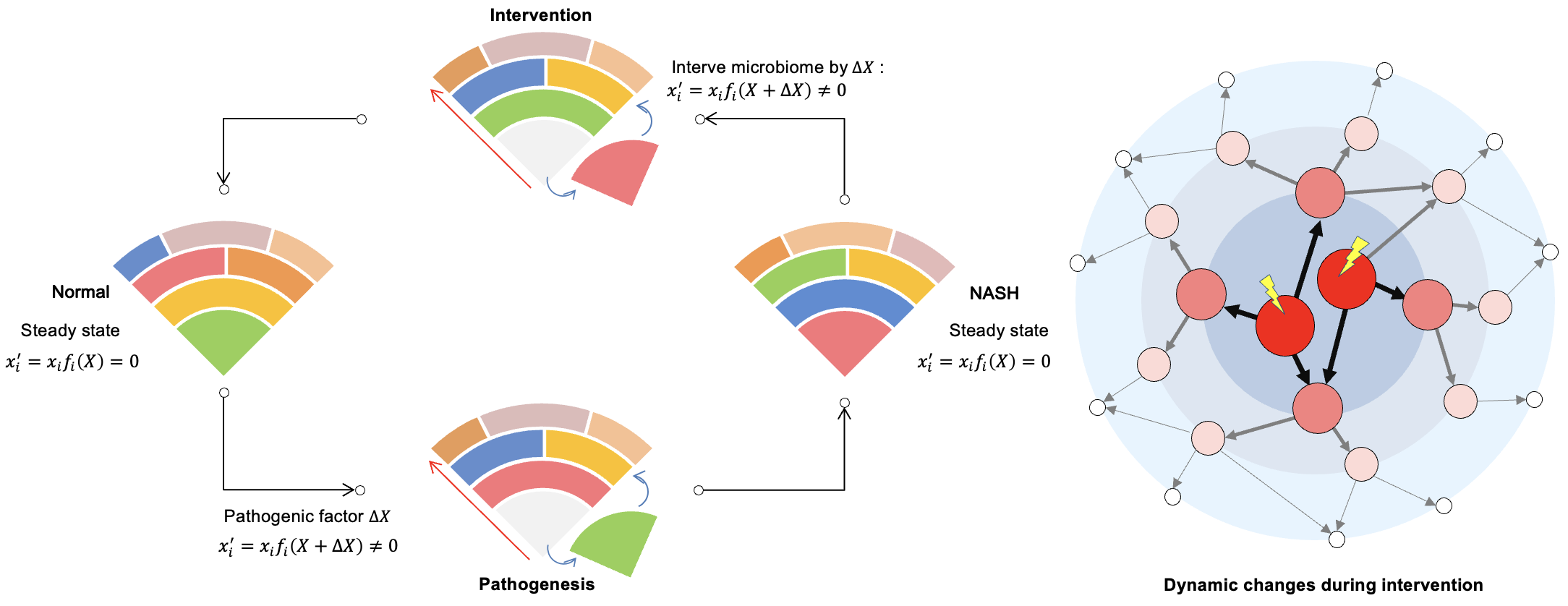


**Figure S8. Schematic diagram of microbial state change and dynamic intervention simulation.**

**Figure S9. The interaction discovery ability of causal inference and correlation in simulated data.** Golden standard dataset was simulated based on generalized Lotka–Volterra (gLV) model. The different proportions of prior knowledge were extracted from the simulated microbial interaction matrix, and then we evaluated the ability of different algorithms for interaction discovery.

**Supplementary Methods**

**Microbial interaction network construction based on causal inference theories**

Based on Robins’ [1] and Pearl’s [2] causal inference theories, and the generalized Lotka–Volterra (gLV) dynamics model for characterizing microbial interactions [3], we designed an algorithm for microbial interaction network construction for cross-sectional data. The specific implementation process is as follows.

**Construction of microbial interaction graphical model based on correlation analysis.** Correlation analyses were performed to construct the graphical model of microbial interactions. In consideration of the characteristics of the microbial sequencing data, SparCC [4] with its outstanding performance for microbial compositional data was chosen to construct the microbial co-occurrence networks with permutation test (1000 permutations). Significant microbial relationships (*P* < 0.01) are extracted to construct the co-occurrence networks, which is the priori knowledge for causal inference analyses.

**Identification of causality between species**. Unlike parameter estimation in machine learning, the core of causal inference theories is "identification". Based on the graphical model, we exhausted all feasible strategies to identify the potential causal relations, such as back-door criterion, front-door criterion, and do-calculus [5].

**Estimation of causal effect sizes**. The strength of the causal effect was estimated with a microbial interaction model. The generalized Lotka–Volterra model (Equation 1), a classical dynamic model, was applied to estimate the causal effect sizes between microbes.

$x_{i}^{'}=x_{i}\left( A_{i}X+r_{i} \right)$ (1)

This gLV equation describes the dynamic changes of taxa abundance regulated by their interactions. Consider a microbial community of N different taxa, where $x_{i}^{'}$ is the growth rate of taxon $i$; $A_{i}=(a_{i1},\ldots,a_{iN})$ is the interaction vector, representing the integrated regulatory effect of all taxa on taxon$i$; $x_{i}$ is the abundance of taxon $i$; $X=(x_{1},\ldots,x_{N})$ is the abundance vector of all taxa; $r_{i}>0$ is the inherent growth rate of taxon $i$.

The gut microbiome of normal or disease state is often stable and resilient [6], which means $x_{i}^{'}\approx0$.

$x_{i}^{'}=x_{i}\left( \sum a_{ij}x_{j}+r_{i} \right)\approx0$ (2)

Where $a_{ij}$ is the interaction coefficient (per capita effect) of taxon $j$ on the growth rate of taxon $i$. In this work, we consider $x_{i}\neq0$ in NASH state. Let $s_{j}=\frac{a_{ij}}{-a_{ii}}$ and $t_{i}=\frac{r_{i}}{-a_{ii}}$. From equation 2, we obtain:

$x_{i}\approx\sum_{j\neq i} {s_{j}x}_{j}+t_{i}$ (3)

This equation implies linear relationship between the abundance of taxa in the steady-state. Combined with the causal relationship identification from step 2, we could estimate the causal effect size between taxa through linear regression.

$x_{i}=\sum_{j\neq i} {s_{j}x}_{j}+t_{i} , \mathrm{where}s_{j} was defined as the causal effect of j\mathrm{on}i$ (4)

The estimation of interaction effect sizes based on the gLV equation allows the subsequent implementation of the dynamic intervention model which is also based on the gLV equation.

**Iterative optimization of causal inference**. The significance of causality was assessed by the permutation test. On the other hand, in order to achieve higher accuracy in causal inference, an iterative optimization strategy was implemented to improve the graphical model, making use of the significant (*P*$\leq$0.01) causal pairs with a certain learning rate (default 0.5). The adjusted graphical model was then subjected to a new round of causal inference. These steps were repeated until convergence.

The above algorithm was developed and implemented based on the DoWhy (<https://microsoft.github.io/dowhy/)> causal inference framework developed by Microsoft. All codes of this algorithm are available at the online python project (https://github.com/tjcadd2020/NAFLD_keystone).

**Keystone species identification based on dynamic intervention simulation**

Keystone species are defined as the species required for maintaining the homeostasis of ecological communities. The alteration of the keystone species could affect the entire community through the interactions among the members of the ecosystem [7]. Therefore, keystone species may be targeted in microbial interventions. We proposed to identify the keystone species with a dynamic intervention simulation (DIS) algorithm with cross-sectional data, as detailed in the following four steps:

**Topological importance evaluation of the species in the interaction networks**. The microbial interaction networks of normal and diseased states were constructed by causal inference, in which the impact of each microbe on the community could be described by network topological importance. Microbial interaction network constructed by causal inference was a directed graph that contains effect intensity and direction. Therefore, the HITS algorithm which computes authorities (Equation 5) and hubs (Equation 6) for nodes in the network was applied [8]. HITS hubs score was used as microbial topological importance score and its significance was evaluated by permutation test with 1000 random networks that have an equal number of nodes and interactions.

$a\left( u \right)=\sum h\left( v \right), a\left( u \right)=\frac{a(u)}{max(a(u))}$ (5)

$h\left( v \right)=\sum a\left( u \right), h\left( v \right)=\frac{h(v)}{max(h(v))}$ (6)

**Dynamic intervention simulation.** With the cross-sectional data, we are able to implement the gLV modeling by focusing on the characteristics of the steady-state of the diseased microbiome (Equation 2). With this approach, we evaluated the impact of the intervention on microbial species at the diseased microbiome by introducing the intervention operation targeting each potential keystone species (Equation 7).

$x_{i}^{'}=x_{i}\left[ \sum a_{ij}{(x}_{j}+\Delta x_{j})+r_{i} \right]$ (7)

Where $\Delta x_{j}$ is the intervention against taxon $j$. Considering that the microbiome is still in steady state at the moment of intervention, $x_{i}^{'}\approx0$. And the effect of the intervention on taxa could be expressed as:

$x_{i}^{'}=x_{i}\sum a_{ij}\Delta x_{j}, \mathrm{where} \Delta x_{j}={-DiffAbun}_{j}$ (8)

Where the ${DiffAbun}_{j}$ indicates the abundance change of taxon $j$ from normal to the disease state.

**Intervention scoring**. In order to comprehensively evaluate the effectiveness of interventions, we designed the intervention score ($IS$) based on the changes of taxa during the intervention.

$IS=sign\left( DiffAbun \right)* sign\left( {-X}^{'} \right)*{HITS\_Score}_{normal}$ (9)

Where the $DiffAbun$ is a vector, representing the abundance change of all taxa from normal to disease. And $X^{'}$ is the vector of abundance change of all taxa after intervention. ${HITS\_Score}_{normal}$ is the topological importances of taxa in the normal state. $IS$ reflects the potential of the intervention to restore the microbiome to normal state.

**Searching for the optimal combinations of the keystone species for microbial intervention**. Iterative Feature Elimination (IFE), a feature selection strategy based on the greedy algorithm [9,10], was used to search for the optimal combination of microbial species for the intervention. The specific operation of the IFE is as follows. First, the intervention score of all taxa, ${IS}_{all}$, is calculated. Then, one taxon was removed from the current combination each time and the intervention score of the remaining taxa combined, ${IS}_{leave-one-out}$, was calculated, and the combination with the highest score, ${argmax(IS}_{leave-one-out})$, was retained for the next removal operation. Repeat the above steps until the optimal combination of the keystone species for microbial intervention was found.

The above algorithm was developed and implemented in python. All codes of this algorithm are available in the online project (https://github.com/tjcadd2020/NAFLD_keystone).

**References**

1. Hernán, Miguel A., James M. Robins. 2019. Causal Inference. Boca Raton: Chapman & Hall/CRC, forthcoming.

2. Pearl, Judea. 2010. “An Introduction to Causal Inference.” The International Journal of Biostatistics 6: Article 7. <https://doi.org/doi:10.2202/1557-4679.1203>

3. Marino, S., N. T. Baxter, G. B. Huffnagle, J. F. Petrosino, P. D. Schloss. 2014. “Mathematical modeling of primary succession of murine intestinal microbiota.” Proceedings of the National Academy of Sciences of the United States of America 111: 439–444. <https://doi.org/10.1073/pnas.1311322111>

4. Friedman, J., E. J. Alm. 2012. “Inferring correlation networks from genomic survey data.” PLoS Comput Biol 8: e1002687. <https://doi.org/10.1371/journal.pcbi.1002687>

5. Shpitser, Ilya. 2012. Graph-based criteria of identifiability of causal questions. Wiley Online Library,

6. Xiao, Y., M. T. Angulo, J. Friedman, M. K. Waldor, S. T. Weiss, Y. Y. Liu. 2017. “Mapping the ecological networks of microbial communities.” Nat Commun 8: 2042. <https://doi.org/10.1038/s41467-017-02090-2>

7. Faust, Karoline, Jeroen Raes. 2012. “Microbial interactions: from networks to models.” Nature Reviews Microbiology 10: 538–550. <https://doi.org/10.1038/nrmicro2832>

8. Kleinberg, J. M. 1999. “Authoritative sources in a hyperlinked environment.” Journal of the Acm 46: 604–632. <https://doi.org/Doi> 10.1145/324133.324140

9. Pang, Herbert, Stephen L George, Ken Hui, Tiejun Tong. 2012. “Gene selection using iterative feature elimination random forests for survival outcomes.” IEEE/ACM Transactions on Computational Biology and Bioinformatics (TCBB) 9: 1422–1431.

10. Lazzarini, N., J. Bacardit. 2017. “RGIFE: a ranked guided iterative feature elimination heuristic for the identification of biomarkers.” Bmc Bioinformatics 18: https://doi.org/10.1186/s12859-017-1729-2
