# Supplementary material for: Targeting keystone species helps restore the dysbiosis of butyrate‐producing bacteria in nonalcoholic fatty liver disease
Source: Imeta. 2022 Nov 16;1(4):e61. doi: 10.1002/imt2.61 (PMC10989787; doi:10.1002/imt2.61)
Supplement: Supplementary file 5 — Supporting information. [file IMT2-1-e61-s003.docx]

**Supplementary Notes**

**Microbial interaction network construction based on causal inference theories**

Based on Robins’ ^1^ and Pearl’s ^2^ causal inference theories, and the generalized Lotka–Volterra (gLV) dynamics model for characterizing microbial interactions ^3^, we designed an algorithm for microbial interaction network construction for cross-sectional data. The specific implementation process is as follows.

**Construction of microbial interaction graphical model based on correlation analysis.** Correlation analyses were performed to construct the graphical model of microbial interactions. In consideration of the characteristics of the microbial sequencing data, SparCC ^4^ with its outstanding performance for microbial compositional data was chosen to construct the microbial co-occurrence networks with permutation test (1000 permutations). Significant microbial relationships (p<0.01) are extracted to construct the co-occurrence networks, which is the priori knowledge for causal inference analyses.

**Identification of causality between species**. Unlike parameter estimation in machine learning, the core of causal inference theories is "identification". Based on the graphical model, we exhausted all feasible strategies to identify the potential causal relations, such as back-door criterion, front-door criterion, and do-calculus ^5^.

**Estimation of causal effect sizes**. The strength of the causal effect was estimated with a microbial interaction model. The generalized Lotka–Volterra model (Equation 1), a classical dynamic model, was applied to estimate the causal effect sizes between microbes.

$x_{i}^{'}=x_{i}\left( A_{i}X+r_{i} \right)$ (1)

This gLV equation describes the dynamic changes of taxa abundance regulated by their interactions. Consider a microbial community of N different taxa, where $x_{i}^{'}$ is the growth rate of taxon $i$; $A_{i}=(a_{i1},\ldots,a_{iN})$ is the interaction vector, representing the integrated regulatory effect of all taxa on taxon $i$; $x_{i}$ is the abundance of taxon $i$; $X=(x_{1},\ldots,x_{N})$ is the abundance vector of all taxa; $r_{i}>0$ is the inherent growth rate of taxon $i$.

The gut microbiome of normal or disease state is often stable and resilient ^6^, which means $x_{i}^{'}\approx0$.

$x_{i}^{'}=x_{i}\left( \sum a_{ij}x_{j}+r_{i} \right)\approx0$ (2)

Where $a_{ij}$ is the interaction coefficient (per capita effect) of taxon $j$ on the growth rate of taxon $i$. In this work, we consider $x_{i}\neq0$ in NASH state. Let $s_{j}=\frac{a_{ij}}{-a_{ii}}$ and $t_{i}=\frac{r_{i}}{-a_{ii}}$. From equation 2, we obtain:

$x_{i}\approx\sum_{j\neq i} {s_{j}x}_{j}+t_{i}$ (3)

This equation implies linear relationship between the abundance of taxa in the steady-state. Combined with the causal relationship identification from step 2, we could estimate the causal effect size between taxa through linear regression.

$x_{i}=\sum_{j\neq i} {s_{j}x}_{j}+t_{i} , \mathrm{where}s_{j} was defined as the causal effect of j\mathrm{on}i$ (4)

The estimation of interaction effect sizes based on the gLV equation allows the subsequent implementation of the dynamic intervention model which is also based on the gLV equation.

**Iterative optimization of causal inference**. The significance of causality was assessed by the permutation test. On the other hand, in order to achieve higher accuracy in causal inference, an iterative optimization strategy was implemented to improve the graphical model, making use of the significant (p$\leq$0.01) causal pairs with a certain learning rate (default 0.5). The adjusted graphical model was then subjected to a new round of causal inference. These steps were repeated until convergence.

The above algorithm was developed and implemented based on the DoWhy (<https://microsoft.github.io/dowhy/)> causal inference framework developed by Microsoft. All codes of this algorithm are available at the online python project (https://github.com/ddhmed/NAFLD_keystone).

**Keystone species identification based on dynamic intervention simulation**

Keystone species are defined as the species required for maintaining the homeostasis of ecological communities. The alteration of the keystone species could affect the entire community through the interactions among the members of the ecosystem ^7^. Therefore, keystone species may be targeted in microbial interventions. We proposed to identify the keystone species with a dynamic intervention simulation (DIS) algorithm with cross-sectional data, as detailed in the following four steps:

**Topological importance evaluation of the species in the interaction networks**. The microbial interaction networks of normal and diseased states were constructed by causal inference, in which the impact of each microbe on the community could be described by network topological importance. Microbial interaction network constructed by causal inference was a directed graph that contains effect intensity and direction. Therefore, the HITS algorithm which computes authorities (Equation 5) and hubs (Equation 6) for nodes in the network was applied ^8^. HITS hubs score was used as microbial topological importance score and its significance was evaluated by permutation test with 1000 random networks that have an equal number of nodes and interactions.

$a\left( u \right)=\sum h\left( v \right), a\left( u \right)=\frac{a(u)}{max(a(u))}$ (5)

$h\left( v \right)=\sum a\left( u \right), h\left( v \right)=\frac{h(v)}{max(h(v))}$ (6)

**Dynamic intervention simulation.** With the cross-sectional data, we are able to implement the gLV modeling by focusing on the characteristics of the steady-state of the diseased microbiome (Equation 2). With this approach, we evaluated the impact of the intervention on microbial species at the diseased microbiome by introducing the intervention operation targeting each potential keystone species (Equation 7).

$x_{i}^{'}=x_{i}\left[ \sum a_{ij}{(x}_{j}+\Delta x_{j})+r_{i} \right]$ (7)

Where $\Delta x_{j}$ is the intervention against taxon $j$. Considering that the microbiome is still in steady state at the moment of intervention, $x_{i}^{'}\approx0$. And the effect of the intervention on taxa could be expressed as:

$x_{i}^{'}=x_{i}\sum a_{ij}\Delta x_{j}, \mathrm{where} \Delta x_{j}={-DiffAbun}_{j}$ (8)

Where the ${DiffAbun}_{j}$ indicates the abundance change of taxon $j$ from normal to the disease state.

**Intervention scoring**. In order to comprehensively evaluate the effectiveness of interventions, we designed the intervention score ($IS$) based on the changes of taxa during the intervention.

$IS=sign\left( DiffAbun \right)* sign\left( {-X}^{'} \right)*{HITS\_Score}_{normal}$ (9)

Where the $DiffAbun$ is a vector, representing the abundance change of all taxa from normal to disease. And $X^{'}$ is the vector of abundance change of all taxa after intervention. ${HITS\_Score}_{normal}$ is the topological importances of taxa in the normal state. $IS$ reflects the potential of the intervention to restore the microbiome to normal state.

**Searching for the optimal combinations of the keystone species for microbial intervention**. Iterative Feature Elimination (IFE), a feature selection strategy based on the greedy algorithm ^9, 10^, was used to search for the optimal combination of microbial species for the intervention. The specific operation of the IFE is as follows. First, the intervention score of all taxa, ${IS}_{all}$, is calculated. Then, one taxon was removed from the current combination each time and the intervention score of the remaining taxa combined, ${IS}_{leave-one-out}$, was calculated, and the combination with the highest score, ${argmax(IS}_{leave-one-out})$, was retained for the next removal operation. Repeat the above steps until the optimal combination of the keystone species for microbial intervention was found.

The above algorithm was developed and implemented in python. All codes of this algorithm are available in the online project (https://github.com/ddhmed/NAFLD_keystone).

**References**

[1] Hernán MA, Robins JM. Causal Inference. *Boca Raton: Chapman & Hall/CRC, forthcoming*. 2019.

[2] Pearl J. Causality: models, reasoning, and inference. *IIE Transactions*. 2002; 34(6):583-589.

[3] Marino S, Baxter NT, Huffnagle GB, Petrosino JF, Schloss PD. Mathematical modeling of primary succession of murine intestinal microbiota. *P Natl Acad Sci USA*. 2014; 111(1):439-444.

[4] Friedman J, Alm EJ. Inferring correlation networks from genomic survey data. *PLoS Comput Biol*. 2012; 8(9):e1002687.

[5] Shpitser I. Graph-based criteria of identifiability of causal questions. Wiley Online Library, 2012.

[6] Xiao Y, Angulo MT, Friedman J, Waldor MK, Weiss ST, Liu YY. Mapping the ecological networks of microbial communities. *Nat Commun*. 2017; 8(1):2042.

[7] Faust K, Raes J. Microbial interactions: from networks to models. *Nature Reviews Microbiology*. 2012; 10(8):538-550.

[8] Kleinberg JM. Authoritative sources in a hyperlinked environment. *J Acm*. 1999; 46(5):604-632.

[9] Pang H, George SL, Hui K, Tong T. Gene selection using iterative feature elimination random forests for survival outcomes. *IEEE/ACM Transactions on Computational Biology and Bioinformatics (TCBB)*. 2012; 9(5):1422-1431.

[10] Lazzarini N, Bacardit J. RGIFE: a ranked guided iterative feature elimination heuristic for the identification of biomarkers. *Bmc Bioinformatics*. 2017; 18(1): 322.
